# Identification of protein N-termini in *Cyanophora paradoxa* cyanelles: transit peptide composition and sequence determinants for precursor maturation

**DOI:** 10.3389/fpls.2015.00559

**Published:** 2015-07-22

**Authors:** Daniel Köhler, Dirk Dobritzsch, Wolfgang Hoehenwarter, Stefan Helm, Jürgen M. Steiner, Sacha Baginsky

**Affiliations:** ^1^Plant Biochemistry, Institute of Biochemistry and Biotechnology, Martin-Luther-University Halle-Wittenberg, BiozentrumHalle (Saale), Germany; ^2^Proteomeanalytik, Leibniz Institute of Plant BiochemistryHalle (Saale), Germany; ^3^Plant Physiology, Institute of Biology, Martin-Luther-University Halle-WittenbergHalle (Saale), Germany

**Keywords:** TAILS, cyanelle, transit peptide, quantitative proteomics, evolution

## Abstract

Glaucophyta, rhodophyta, and chloroplastida represent the three main evolutionary lineages that diverged from a common ancestor after primary endosymbiosis. Comparative analyses between members of these three lineages are a rich source of information on ancestral plastid features. We analyzed the composition and the cleavage site of cyanelle transit peptides from the glaucophyte *Cyanophora paradoxa* by terminal amine labeling of substrates (TAILS), and compared their characteristics to those of representatives of the chloroplastida. Our data show that transit peptide architecture is similar between members of these two lineages. This entails a comparable modular structure, an overrepresentation of serine or alanine and similarities in the amino acid composition around the processing peptidase cleavage site. The most distinctive difference is the overrepresentation of phenylalanine in the N-terminal 1–10 amino acids of cyanelle transit peptides. A quantitative proteome analysis with periplasm-free cyanelles identified 42 out of 262 proteins without the N-terminal phenylalanine, suggesting that the requirement for phenylalanine in the N-terminal region is not absolute. Proteins in this set are on average of low abundance, suggesting that either alternative import pathways are operating specifically for low abundance proteins or that the gene model annotation is incorrect for proteins with fewer EST sequences. We discuss these two possibilities and provide examples for both interpretations.

## Introduction

Photosynthetic organisms originated from a primary endosymbiotic event in which a free-living ancient cyanobacterium was taken up by a mitochondria-containing eukaryotic host cell and modified to evolve into plastids. Subsequent to primary endosymbiosis, three distinct evolutionary branches emerged and evolved into today's rhodophyta, chloroplastida and glaucophyta (Bhattacharya et al., 2004; Adl et al., 2005). Most research has been devoted to members of the chloroplastida branch, since it represents around 99% of plant biomass on the mainland and performs most of the photosynthetic activity on this planet. However, recent sequencing efforts established the genome sequence of *Galdieria sulphuraria* and *Cyanophora paradoxa* as representatives for rhodophytes and glaucophytes, making comparative sequence analyses and functional genomics approaches possible (Price et al., 2012; Schönknecht et al., 2013). With proteomics tools at hand, the evolutionary divergence of fundamental plastid processes can now be studied at the protein level, which has the advantage over comparative genomics that such analyses are closer to gene function.

The key to establishing an endosymbiosis is the distribution of tasks between the cell organelles, and their coordination by the nucleus as the central gene expression system. Thus, the endosymbiont has lost much of its original genome coding capacity to the nucleus (Martin et al., 2002). Since plastids are the site of many complex metabolic processes, there is clearly a need for the re-import of nuclear encoded plastid proteins from the cytosol in a specific and regulated manner. At the outer and inner plastid envelope membranes, a specialized import machinery has evolved that comprises high molecular mass protein complexes designated as translocases at the outer (TOC) and inner (TIC) chloroplast envelope membrane (Agne and Kessler, 2009). A basic set of common TOC and TIC components is maintained in all descendants from primary endosymbiosis, representing most likely the basic functional module in plastid protein import (Steiner and Löffelhardt, 2005). This supports the single origin of primary plastids and furthermore suggests that plastid protein import has been established prior to the divergence of the three distinct evolutionary branches.

Therefore, basic characteristics of import specificity must have been in place prior to the evolutionary branching. Proteins that are destined for plastids possess an N-terminal transit peptide that is cleaved off after plastid protein import by a processing peptidase. At present, most of the information on transit peptide design is available for the chloroplastida, mostly Arabidopsis, rice, and Chlamydomonas (Patron and Waller, 2007; Chotewutmontri et al., 2012). In the case of Arabidopsis, an N-terminal HSP70-binding motif enriched in hydrophobic amino acids is required for protein translocation (Chotewutmontri et al., 2012). Chloroplastida transit peptides are generally devoid of acidic amino acids and enriched in hydroxylated amino acids. Serine is the most frequently occurring amino acid in Arabidopsis transit peptides, while it is alanine in rice and Chlamydomonas (Kleffmann et al., 2007; Patron and Waller, 2007; Zybailov et al., 2008). Surprisingly, apart from a modular structure, transit peptides are not well conserved and only few functional modules were identified that are involved in protein translocation (Lee et al., 2008).

Initial attempts were made to compare chloroplastida transit peptides with those of rhodophyta and glaucophyta but these analyses were hampered by the constrained datasets available for the latter two evolutionary branches. The most striking feature of their transit peptides is the occurrence of a phenylalanine at the N-terminus, that is prevalent in most known rhodophyte transit peptides (85% carry an F in their N-terminal region) and in all known glaucophyta transit peptide (100%, however only 23 sequences were considered) (Patron and Waller, 2007). The functional importance of F is apparent from import experiments that showed that chloroplastida transit peptides are only functional in glaucophytes when phenylalanine is added to the N-terminal region (Steiner et al., 2005b). Apparently, phenylalanine in cyanelle transit peptides is an ancestral feature that was lost during the evolution of the chloroplastida branch.

Since glaucophytes were the first lineage to diverge after plastid primary endosymbiosis (Qiu et al., 2012) the composition of the cyanelle proteome and the mechanisms for assembling it are of particular interest for comparative analyses. We report here a targeted analysis of N-terminal peptides in cyanelle proteins from *Cyanophora paradoxa* using a proteomics method called “terminal amine labeling of substrates” (TAILS) (Doucet et al., 2011). This allowed us to assess protein N-termini as they accumulate *in vivo* and identify sequence requirements for processing peptidase cleavage and functional protein import.

## Materials and methods

### Biological material

*Cyanophora paradoxa* LB555UTEX was grown photo-autotrophically at 26°C under continuous fluorescent white light using a liquid mineral medium continuously gassed with a mixture of 5% (v/v) CO2 in air as previously described (Mucke et al., 1980).

### Isolation of intact and periplasm-free cyanelles

Import-competent cyanelles were isolated according to Steiner et al. (2000). In brief, cultures in exponential growth phase were centrifuged at 3500 × g for 5 min at room temperature. The blue-green pellet was then gently resuspended at 4°C using a buffer containing 25 mM HEPES/NaOH pH 7.6, 2 mM EDTA and 0.35 M Sucrose. *C. paradoxa* cells were broken in a Waring blender 5 × 1 min at maximal speed with 1 min cooling on ice in between. Intact cyanelles were sedimented by centrifugation (2000 × g, 5 min, 4°C) and resuspended twice to remove residual cell debris and contaminant cytosolic proteins. For preparation of periplasm-free cyanelles, plastids were treated with 1% Triton X-100 for 10 min on ice, prior to centrifugation.

### Extraction of proteins and enrichment of N-terminal peptides

For the analysis reported here we used total *C. paradoxa* celles as well as isolated cyanelles as biological material. For total protein extraction a buffer containing 100 mM HEPES/NaOH (pH 7), 4% (w/v) SDS, 1 mM PMSF and 0.1% protease inhibitor cocktail was used for both preparations (Sigma-Aldrich). The enrichment of protein N-termini from *C. paradoxa* samples was performed by terminal amine isotopic labeling of substrates (TAILS) as described by Köhler et al. (2015) based on the protocol of Doucet et al. (2011). The workflow was performed with 100 μg of each sample. The protein-N-termini were chemically blocked by dimethylation. The chemically modified proteins were digested with trypsin and the resulting peptides carrying unblocked primary amines covalently bound to a polymer. Removal of the polymer by filtration results in a sample enriched in N-terminal peptides, which was analyzed by mass spectrometry after sample desalting.

### Setup for mass spectrometric measurements of TAILS samples

The desalted and completely dried samples were resuspended in water with 2% acetonitrile and 0.1% formic acid. Samples were separated by liquid chromatography using C18 columns from Proxeon (guard column: 2 cm, ID 100 μm, 5 μm, analytical column: 10 cm, ID 75 μm, 3 μm). For separation, a gradient consisting of water with 0.1% formic acid (A) and acetonitrile with 0.1% formic acid (B) was used (method: 0-150 min 5-40% B, 150-160 min 40-80% B, 160-170 min 80% B). The samples were analyzed on an LTQ Orbitrap Velos (*Thermo Scientific*), precursor ion detection was done in an m/z-range from 400-1850 and the 20 most intensive signals were used for MS2.

### TAILS data processing and analysis

The MS-raw-data were processed as previously described (Köhler et al., 2015) using Proteome Discoverer 1.2 (Thermo Scientific) and MaxQuant (Cox and Mann, 2008). We used a precursor mass tolerance of 7 ppm and a fragment ion mass tolerance of 0.5 Da with a maximal number of 3 missed cleavages. In all searches, semi tryptic peptides were accepted. MaxQuant searches were performed with a protein FDR of 0.01 and ProteomeDiscoverer searches with a strict FDR of 0.01 and a relaxed FDR of 0.05. The *C. paradoxa* protein database was retrieved from the “Cyanophora Genome Project” (Price et al., 2012). Searched N-terminal modifications were “dimethylation” or “acetylation.” Further, modifications allowed were dimethylation on lysines, carbamidomethylation of cysteines and methionine oxidation. For further analysis we only used peptides with a tryptic C-terminus (Arg, Lys) and where the assigned proteins in the *Cyanophora paradoxa* protein database contained a start-methionine. Cyanelle localized proteins were identified from the cyanelle preparation performed here, by cross-comparing the data against recent proteomics results (Facchinelli et al., 2013), and by homology to proteins in the Arabidopsis chloroplast proteome reference table (Reiland et al., 2009; van Wijk and Baginsky, 2011).

### Nano-LC separation, HD-MS^E^ data acquisition and protein identification/quantification

LC separation (140 min gradient) and HD-MS^E^ data acquisition was performed using 1 μl of the digested total protein samples of TritonX-100 treated or untreated cyanelles on an ACQUITY UPLC System coupled to a Synapt G2-S mass spectrometer (Waters, Eschborn, Germany). MS acquisition was set to 50–2000 Da. Data analysis was carried out by ProteinLynx Global Server (PLGS 3.0.1, Apex3D algorithm v. 2.128.5.0, 64 bit, Waters, Eschborn, Germany) with automated determination of chromatographic peak width as well as MS TOF resolution. Lock mass value for charge state 2 was defined as 785.8426 Da/e and the lock mass window was set to 0.25 Da. Low/high energy threshold was set to 180/15 counts, respectively. Elution start time was 5 min, intensity threshold was set to 750 counts. Databank search query (PLGS workflow) was carried out as follows: Peptide and fragment tolerances was set to automatic, two fragment ion matches per peptide, five fragment ions for protein identification, and two peptides per protein. Maximum protein mass was set to 250 kDa. Primary digest reagent was trypsin with one missed cleavage allowed. According to the digestion protocol, fixed (carbamidomethyl on Cys) as well as variable (oxidation on Met) modifications were set. The false discovery rate (FDR) was set to 4% at the protein level. MS^E^ data were searched against the same *C. paradoxa* database as for the TAILS data analysis, and rabbit glycogen phosphorylase B (P00489) was used as internal quantification standard. Quantification was performed based on the intensity of the three most abundant proteotypic peptides.

### Availability of mass spectrometry data

All mass spectrometry data have been deposited to the ProteomeXchange Consortium (http://proteomecentral.proteomexchange.org) via the PRIDE partner repository with the submission number PXD002187.

## Results and discussion

### Identification of cyanelle proteins and their transit peptides

We analyzed the composition and the processing site of *Cyanophora paradoxa* cyanelle transit peptides by a specialized proteomics method for the specific and sensitive identification of protein N-termini. Using terminal amine isotopic labeling of substrates (TAILS) we enriched N-termini of proteins isolated from cyanelles and full cells from a *Cyanophora paradoxa* culture. The TAILS method works by blocking the primary amine of the protein N-terminus by dimethylation prior to tryptic digest (Boersema et al., 2009; Doucet et al., 2011; Huesgen et al., 2013). Trypsin digestion generates new “unblocked” N-termini that are subsequently coupled to a high molecular weight dendritic hyperbranched polyglycerol-aldehyde (HPG-ALD) polymer with their primary amines. The polymer with the bound internal peptides is removed, while the dimethyl-labeled N-terminal peptides remain in the sample. Thus, native *in vivo* N-termini of *C. paradoxa* proteins are enriched by a subtractive approach and can be unambiguously identified by their dimethyl label in mass spectrometry.

**Figure 1:**
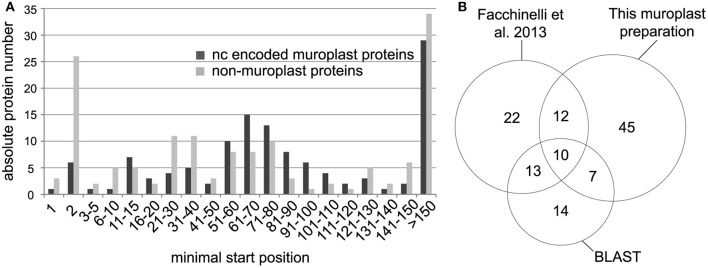
**(A)** Minimal peptide position of peptides identified from nucleus encoded cyanelle proteins (black bars) and non-cyanelle proteins (gray bars). Peptides are sorted into bins representing the amino acid position number of the peptide start. **(B)** Numbers of identified nucleus encoded cyanelle proteins. Assignment to the cyanelle was based on the detection here, a comparison with a recent cyanelle proteome study and a BLAST search against the Arabidopsis chloroplast reference proteome.

We identified non-redundant N-terminal peptides for 303 proteins in an Orbitrap Velos (Figures 1A,B, Supplemental Table S1). Plastid proteins were identified from the cyanelle preparation performed here and from the complete cell preparation by cross comparing the data to a recent cyanelle proteome study (Facchinelli et al., 2013) and by homology to proteins in the *Arabidopsis thaliana* plastid proteome map (Reiland et al., 2009; van Wijk and Baginsky, 2011). For the homology searches we accepted the best Arabidopsis hit at a threshold of e-10, and extracted chloroplast proteins from the list of homologs with a chloroplast proteome reference table (Reiland et al., 2009). With these criteria, we identified 123 nucleus encoded cyanelle proteins in the TAILS dataset (Supplemental Table S1) of which 74 identifications originated from the cyanelle preparation performed here (Figure 1B). The minimal start position of peptides in the identified nucleus encoded proteins is presented in a histogram in Figure 1A, separately for nucleus encoded cyanelle and non-cyanelle proteins (Figure 1A). As reported for other systems, the largest bin for non-cyanelle proteins is that of position “2,” revealing N-terminal methionine excision as an important posttranslational process in *Cyanophora paradoxa* (Giglione et al., 2015). A local maximum at starting positions 21–40 and 51–80 in the set of non-cyanelle proteins comprises most likely mature mitochondrial proteins and so far unknown cyanelle proteins that were generated by target peptide cleavage following import. Indeed, 13 out of 53 proteins falling into these bins carry a phenylalanine within the N-terminal 10 amino acids and therefore fulfill a basic requirement for cyanelle targeted proteins (Supplemental Table S1). Proteins with the start position >150 are most likely derived from proteolytic events that occur during protein activity control and breakdown.

### Characteristics of *Cyanophora paradoxa* transit peptides

Together, 77 nucleus encoded plastid proteins were identified matching the minimal starting range 11–110. (Table 1, Supplemental Table S1). Compared to the set of 136 Arabidopsis proteins identified with the same method (Köhler et al., 2015), Cyanophora transit peptides are slightly longer with a median length of 63 amino acids compared to 55 amino acids for Arabidopsis, however, this difference is not significant (*p* = 0.2, two-tailed Welch's-test). Transit peptides of higher plants possess several distinct modules that are combined and exchanged to result in tissue- and age-specific plastid protein import, a complexity that has been summarized elegantly in the “M and M” model (Li and Teng, 2013). Tissue-specificity of transit peptides is irrelevant for unicellular organisms and it was therefore expected that cyanelle transit peptides are rather shorter than those of Arabidopsis, e.g., more similar in size to those of Chlamydomonas (around 32 amino acids) (Patron and Waller, 2007). One possible explanation for the lengths of Cyanophora transit peptides is the peptidoglycan wall between the outer and inner envelope membrane that must be crossed by imported proteins.

**Table 1:** **List of all nucleus encoded cyanelle proteins that match the minimal starting range 11–110 as identified by TAILS**.

**CDS-ID**	**Peptide ID**	**First 10 amino acids**	**Detected minimal position**	**Cyanelle localisation determined by**		
				**published**	**BLAST**	**This study**
10247_cds:3280	AEGPAPNVNPLTGAPR	MA**F**VVTLPSG	75	−	−	+
10778_cds:3661	AGTAAGASAAVAEAR	MRRVAALLSA	14	−	−	+
10872_cds:3470	AGKQITVGVIGAGR	MAA**F**TTPLVL	79	−	−	+
12207_cds:4390	TGGASEPGPGAYTIDVGK	MGEGTAAHGG	59	−	−	+
12588_cds:4370	SINVPAVMSDVNAAAK	MRDTTDSDTG	66	−	−	+
12656_cds:4591	AGEEASMAHYAAPAVGALR	MVQEGKLAPE	64	−	−	+
37200_cds:4999	EEVSAAKKELAER	MQRLYRI**F**AI	84	−	−	+
37396_cds:7195	LVNDSSPDPSLQR	MQP**F**DEDVML	11	−	−	+
38265_cds:8081	TARWRASSVASEANGQR	MQLPPGAAPA	90	−	−	+
38986_cds:8738	FAGDALVAFVAAGELEGGGR	MLRLSSTLNA	29	−	−	+
39427_cds:8972	AGAEGGLEPDR	MA**F**STSGLGL	81	−	−	+
52758_cds:10110	AELVPLDYGAIR	MAALPVVGQI	16	−	−	+
52931_cds:10073	VNAATGDAAANRAR	MAVGAGSLNT	82	−	−	+
54348_cds:11201	EPAQPSPEELAAREAER	MAEAGEASPA	91	−	−	+
6638_cds:99/60	GLEMIASENFTSR	MSAPRRQLPM	37	−	−	+
6977_cds:523/2	AADSGAGAGGAAAGPR	MAMQRDGSLP	40	−	−	+
7258_cds:763/1	EAATSPGPSASAAPAAGGR	MDTA**F**ATGAP	65	−	−	+
7675_cds:1094	AGSSSAAPSPAK	MDRD**F**NGARN	63	−	−	+
8688_cds:1951	AAAPLEEDYTATPSGLR	MIPAAA**F**VPP	84	−	−	+
9509_cds:2612	ALDATKDAVGSAAR	MAPA**F**VVAAP	84	−	−	+
25545_cds:5400	GVLKVFLENVIR	MSGRGKGGKG	57	+	−	−
37212_cds:6941	KSGEATGLDVLIVR	MNA**F**VAPAPL	106	+	−	−
39194_cds:8857	AVPLLAYKPKTTNTR	MA**F**VTPVPAV	51	+	−	+
54421_cds:11004	SRVPVELIFDQGFGDVFVTR	MSPAVSPKSS	62	+	−	−
56196_cds:11903	NSAAGASDGSFNPYR	MAYISSLPGS	70	+	−	+
8154_cds:1523	SGPAGDPRFPVMSGEVPQDLSVYDKDGVAR	MTAVLPTTQT	110	+	−	−
8403_cds:1901	SGGITKSEPTFGGIETPAGAEVEIIR	MAQQITGVNP	12	+	−	−
8517_cds:2013	SIPIIEFAPKTTNTR	MAA**F**ATPIAV	52	+	−	+
8620_cds:2109	KKGSGAGRPTGAPGGSMPPMPSQAAAGDLPVIYIFAR	M**F**SPPRSDD**F**	58	+	−	−
8899_cds:2361	DTSGSAIKRITNPSSSPIAGDNR	MAS**F**VAATGL	43	+	−	+
38654_cds:7924	TDHPDYLGSLTQASVVR	MSSAETVL**F**S	99	−	−	+
54708_cds:11657	AKTADAPAYAR	MPPKRSSKRG	26	−	−	+
25596_cds:5692	NAEADKLTEATAR	MA**F**TVAPVAP	61	+	−	+
37768_cds:7858	VATDKITPLPMKGNAPLSDADPEVFDLIER	MAA**F**ASSIVA	64	+	−	+
38084_cds:7872	AAQLGVALTNGNAR	MVSPVVKRPI	106	+	−	−
39122_cds:8823	GVDLEAGGR	MWPLGGYGPA	29	+	−	−
8923_cds:2292	SGFGREESIIADMSLADYGR	MALQLNLKAK	18	+	−	−
12743_cds:4543	ASAKPDTGPSQGQFSR	MLRAAAAARR	31	−	−	+
40999_cds:9485	ATNAVATPAYSELSSVPLYQQEKKIER	MSATMLRLAR	31	+	−	−
7974_cds:1883	SSTSQPLASVVTGRK	MLSLRRLAPS	22	+	−	−
9664_cds:3056	GATVVDTGAPIMIPVGR	MSTPAKAAPA	79	+	−	−
10978_cds:3594	EAPAESSDAEATDR	MESC**F**VAGLG	71	−	+	+
11467_cds:3786	ASASAPKLEVENVVIIGSGPAGLTSAIYTAR	MLG**F**AVSS**F**P	95	−	+	−
13287_cds:4604	ATATAAQGVLPR	MAAVGTSLLR	14	−	+	−
26296_cds:6567	SLANDSSAGNDQLDIRR	MA**F**VGTPVAA	101	−	+	+
37389_cds:7124	EGLTYDQLNSANYLKLKGTGISNTCPTLSTSAR	MA**F**VNTVPV**F**	85	−	+	+
38529_cds:8386	SSSAAAADAGPSQR	MPS**F**VTGTAA	55	−	+	+
38915_cds:8582	SSQQVKDASSTESQSATAR	M**F**STAPSSAS	44	−	+	−
39038_cds:8649	SANNLPPTYTGVSR	MA**F**S**F**GIAAA	75	−	+	+
39838_cds:8861	GLGNSFGHLFR	M**F**VRSARHEA	38	−	+	−
53054_cds:9835	QFSEVYVEVAKPLGVTFEEGPDGR	MA**F**IAPCPAI	69	−	+	−
53171_cds:10566	ATQAVASSASICASAAPR	MA**F**VLNVPTR	14	−	+	+
53311_cds:10649	SAASPSSEDILIVGAGVLGSR	M**F**AVCLSPAA	70	−	+	−
7674_cds:1262	AIQPAAVDLGMPLDVLSR	MAA**F**VASIPA	62	−	+	−
8074_cds:1451	SLIEQQEEAEKAAR	META**F**ATAAL	73	−	+	+
8340_cds:1819	SVEQLQKVLEEQKALPR	MQWLRAAATA	97	−	+	−
8437_cds:1947	EAGGGSSPASVSPLDGAEDDGLAVRR	M**F**VSLIPSAL	57	−	+	−
10670_cds:3614	EAAPAQQKGIEEHVATDVVPDSFLR	MESA**F**LVPAP	69	+	+	−
11368_cds:3893	ANPAVASAACSSVAPARAIR	MA**F**TLAPVAA	16	+	+	−
25517_cds:5494	AVEIAAATVKELR	MA**F**VNVVSQA	15	+	+	+
25643_cds:5691	ATAAMTPVDDK	MSA**F**VVPVPS	99	+	+	+
37061_cds:6844	SAASDDVPDMGKR	MA**F**TTTAVVA	61	+	+	+
37854_cds:7838	EEEEAAPAEKVEKKAAAPKPFSVPTLNLNAPTPIFGGSTGGLLR	MNA**F**VASVAP	54	+	+	−
38681_cds:8352	SSVAAESSEQTSQPATTSSKTER	MA**F**LGSAVAG	74	+	+	−
40175_cds:9516	RMSAITAEPVGTPETLEYR	MEVA**F**AVPLA	73	+	+	+
52898_cds:9949	QAEDATAEIADTR	MAAAPAVVRA	11	+	+	+
52975_cds:10182	EEQSSPSAAGKSDAESDASGVSR	MQAD**F**GGGQS	59	+	+	−
53396_cds:10332	ASTEVVTGDDMKGETFTR	MA**F**ATVAPVA	77	+	+	+
53411_cds:10720	AKDAVKPFYDDAFIGHLSTPISNSSAVNGLLANLPAYR	MA**F**ITAIPAV	55	+	+	−
53536_cds:10786	GGLDLGSLSPAKGSNR	MA**F**S**F**APVAA	69	+	+	+
54611_cds:11381	SATGFAAAIADKSVLDFDSKIFTKEVIQLADTQESIVR	MSSA**F**ATVAP	93	+	+	−
7394_cds:1013	ANPADAPPPPFGAGPKEDALEGFVSSIPGSLR	MQTENSTAPA	77	+	+	+
7699_cds:1095	SASGSAASKDAVIDQFTSNVMTTYAR	MESVAA**F**ACA	90	+	+	−
8081_cds:1633	SFSTAKSDEIFKR	MDTA**F**ALAVP	61	+	+	+
8139_cds:1758	EDEQPKPSVPEPDVSGNALGLGQLQNVFTR	MAA**F**VAGAQA	74	+	+	−
8721_cds:2147	STSIVTTTKHTEIVELLVPESQR	MA**F**VAPIVAV	76	+	+	−
8725_cds:2214	SADSGSLLDNIPFR	MAA**F**ACGVPV	71	+	+	−

*The proteins are represented by their CDS-Identifier and the identified peptide representing the minimal start identified for the protein. Phenylalanine (F) within the first 10 amino acids is highlighted and underlined. The data underlying the assignment of proteins as cyanelle proteins are indicated with a plus (+)*.

“*published”: Cyanelle proteins previously identified by proteomics (Facchinelli et al., 2013)*.

“*BLAST”: C. paradoxa proteins identified as cyanelle proteins by BLAST searches against an A. thaliana chloroplast proteome reference table (van Wijk and Baginsky, 2011)*.

“*This study”: Proteins identified by mass spectrometry in samples of purified cyanelles in this study*.

**Figure 2:**
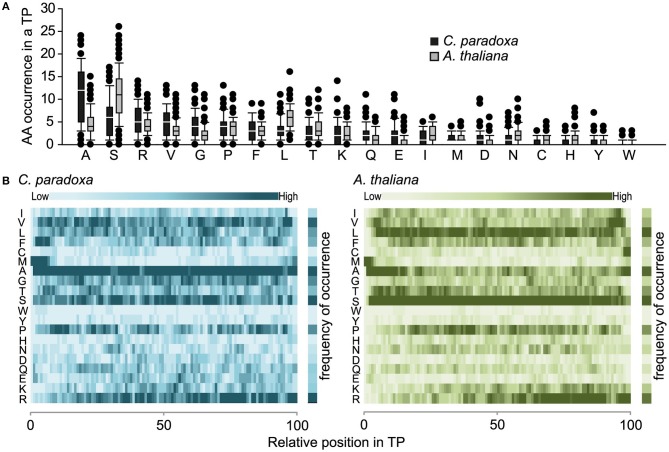
**(A)** Amino acid composition of transit peptides identified from Arabidopsis and Cyanophora. For every amino acid, the average occurrence in transit peptides is provided along with the standard deviation. **(B)** Topology heat map of amino acid distribution in transit peptides of *Arabidopsis thalia*na and *Cyanophora paradoxa*. The number of amino acids in every transit peptide was set to 100% and the relative position of amino acids in the transit peptide was calculated on this basis. The darker the color the higher the frequency of occurrence.

The overall amino acid composition is largely similar except that serine is prevalent in Arabidopsis and alanine in *Cyanophora paradoxa* transit peptides (Figures 2A,B). In this respect, the latter are similar to transit peptides of rice and Chlamydomonas, while Arabidopsis transit peptides are exceptional (Kleffmann et al., 2007; Patron and Waller, 2007). Because alanine tends to form alpha-helices, it is conceivable that alpha-helical structures might be functional determinants of *Cyanophora paradoxa* transit peptides (Otaki et al., 2010). However, alanine is overrepresented along the entire transit peptide (Figure 2B), speaking against the formation of specific regulatory helical structures. At present, only limited information is available for the role of secondary structure elements in precursor recognition and organellar import and most analyses of transit peptide secondary structures were performed under non-physiological conditions (von Heijne et al., 1989; Franzen et al., 1990; Bruce, 2001). The situation is further compounded by the fact that most transit peptides will form a random coil under aqueous conditions, while functionally important structures only form upon contact with lipid-containing hydrophobic surfaces at the envelope membranes (Wienk et al., 1999; Bruce, 2001; Ambroggio et al., 2007).

### *Cyanophora paradoxa* transit peptides have a modular structure

**Figure 3:**
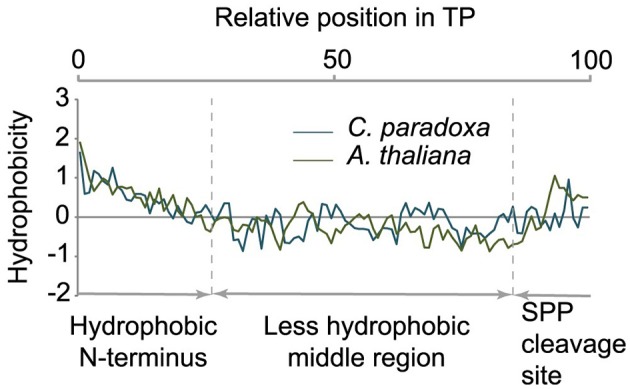
**Tripartite structure of transit peptides from ***Cyanophora paradoxa*** and ***Arabidopsis thaliana*****. The average hydropathy trend of all detected transit peptides is shown. The relative position in the transit peptides is given in the top panel as percentage. According to the hydropathy trend, transit peptides can be divided into three modules as indicated in the panel below.

Transit peptides of both Cyanophora and Arabidopsis have a tripartite modular structure consisting of a hydrophobic N-terminus, a less hydrophobic middle region and a C-terminal region that contains the cleavage site for the processing peptidase (Figure 3). The hydrophobic N-terminal region is required for interaction with cytosolic HSP70 (Ivey et al., 2000; Chotewutmontri et al., 2012), which is essential for plastid protein import (Pilon et al., 1995). One prevalent amino acid in the N-terminus of cyanelle transit peptides is a phenylalanine residue that occurs within the first 10 amino acids (Table 1, Figure 2B). N-terminal phenylalanine is detectable in 49 out of 77 cyanelle transit peptides identified here, thus, phenylalanine is clearly the most distinguishing feature of cyanelle transit peptides compared to those of the chloroplastida branch (i.e., rice, Arabidopsis and Chlamydomonas, Figure 2B) (see discussion below).

In addition to phenylalanine, valine is overrepresented in the N-terminal region of cyanelle transit peptides (Figure 2B) while it is leucine in Arabidopsis transit peptides. Together these three amino acids (V, L, F) together with the weakly hydrophobic amino acid alanine establish the hydrophobicity of the N-terminal module in the transit peptides of both organisms (Figures 2A,B). The hydrophobic N-terminal part and the less hydrophobic middle region of cyanelle transit peptides are separated by several proline residues between amino acid positions 10–20. Proline is present in loops and in regions between secondary structures (Otaki et al., 2010). It is conceivable that the frequent occurrence of proline up to amino acid 20 constitutes a structural separation of the N-terminal 10–20% of the cyanelle transit peptide from the rest. Bruce (2001) hypothesized that such proline containing regions may be necessary to separate two functional regions from each other or function as motifs by themselves (Bruce, 2001). Following the hydrophobic N-terminal region, hydrophobicity decreases as the amount of charged amino acids increases in both organisms. In the identified cyanelle transit peptides, this region contains on average more acidic amino acids than in Arabidopsis (Figure 2B). It is possible that these are required for the efficient transfer of proteins through the periplasm, but functional data on the translocation process are currently not available.

### The processing peptidase cleavage site

**Figure 4:**
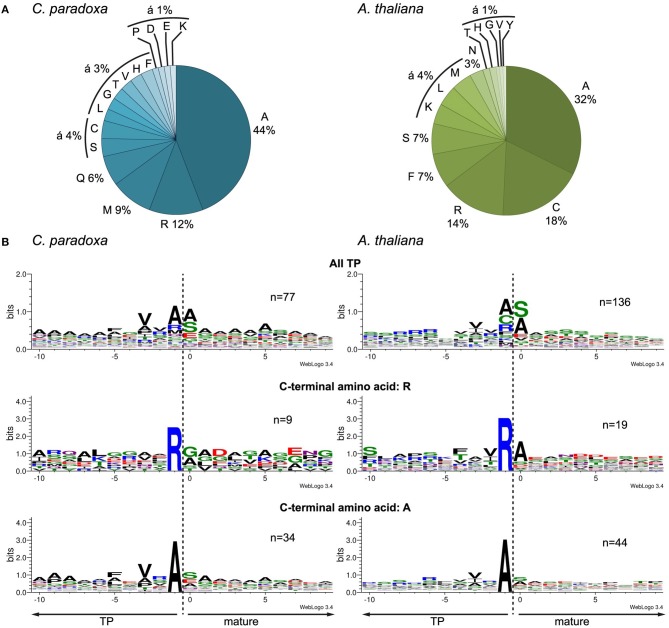
**Characteristics of the transit peptide cleavage site in Arabidopsis and Cyanophora (A) Frequency of amino acid occurrence in the -1 position C-terminal of the cleavage site**. **(B)** Alignment of the identified N-terminus with 10 amino acids up- and downstream of the anticipated cleavage site. First panel, all identified transit peptide, second panel transit peptides with a C-terminal arginine, and third panel transit peptides with a C-terminal alanine. The alignment was performed with WebLogo 3 (Crooks et al., 2004).

We aligned the identified N-termini of cyanelle proteins with WebLogo 3 (Crooks et al., 2004) and ICE Logo (Colaert et al., 2009) using the 10 amino acids up- and downstream of the most N-terminal amino acid. Both procedures produced essentially identical alignments and we therefore present only the WebLogo 3 results. This alignment revealed a local “bit” maximum (y-axis) N-terminal to the processing site resulting mostly from the enrichment of valine, alanine, and arginine within the positions -3 to -1 relative to the cleavage site (Figures 4A,B). The occurrence of alanine as C-terminal amino acid is coupled to the occurrence of valine in the -3 position (Figure 4B). The transit peptides with a C-terminal arginine show no further “bit” maximum (Figure 4B). We can exclude that these peptides are artifacts from the tryptic digest because we accepted only peptides that were identified with a dimethyl-label or an acetyl-group at their N-terminus (the latter modification occurs *in vivo* and blocks the N-terminal dimethyl labeling). Thus, our data suggest that arginine alone is capable of orchestrating the processing peptidase cleavage. The composition of the cleavage site in Arabidopsis transit peptides identified with TAILS is similar, especially the enrichment of valine, arginine, and alanine close to the processing site (Figure 4B). Additionally, Arabidopsis transit peptides are enriched for isoleucine residues that have most likely the same function as valine in Cyanophora (Köhler et al., 2015).

**Figure 5:**
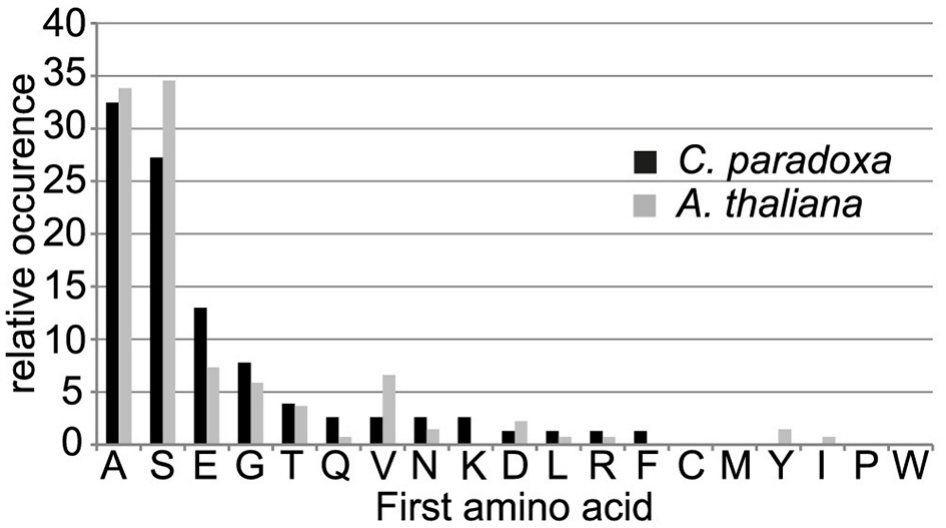
**Frequency of occurrence of amino acids at the most N-terminal position in the mature protein**.

The processing peptidase cleavage releases an N-terminal amino acid, the nature of which may have regulatory impact on protein stability (Apel et al., 2010). Interestingly, the occurrence of amino acids at the N-terminus is similar for both Arabidopsis and Cyanophora (Figure 5) with the majority of proteins carrying serine or alanine in the first position. The greatest difference between these two organisms is the relatively high frequency of cysteine residues around the cleavage site in Arabidopsis that is missing in Cyanophora (Figure 4A). Although transit peptide cleavage prediction programs predict cleavage C-terminal of cysteine (Emanuelsson et al., 2000), it does not occur as N-terminal amino acid in the mature protein (Figure 4). This observation can be explained by postulating an additional cleavage by an intermediate cleavage peptidase (ICP55) (Huang et al., 2015) that removes destabilizing residues from the N-terminus of mature proteins in Arabidopsis. At present we do not have any indication that a similar cleavage occurs in cyanelles.

### Quantitative proteomics supports the prevalence of phenylalanine, but reveals some deviation for low abundance proteins

We next generated a quantitative proteome dataset for isolated cyanelles to set the above-described observations into a biological context. In order to focus the analysis on transit peptides in proteins that have crossed two membranes, we generated two different quantitative proteomics datasets, (i) one set from isolated cyanelles including the periplasmic space and (ii) another set from isolated cyanelles from which the periplasm was removed by Triton X-100 washes. Absolute protein quantification was performed by HD-MS^E^ as described previously (Helm et al., 2014). All data were acquired in three replicate measurements and only proteins consistently identified and quantified in at least 2 out of 3 replicates in both preparations were considered for further analyses. To identify periplasmic proteins and to remove them from further analysis, we included only those proteins in our analysis whose abundance is less than 2-fold different between the two preparations. With this constraint, the final dataset comprises 276 quantified proteins (Supplemental Table S2). The most abundant proteins are cyanelle-encoded phycocyanin subunits, phycobilisome linker proteins and other photosynthetic proteins, while several uncharacterized proteins, as well as enzymes involved in amino acid and nucleotide metabolism are the least abundant proteins in this dataset (for a detailed list see Supplemental Table S2).

Altogether, 19 proteins with an obviously incomplete gene model (no start methionine) and the remaining 70 cyanelle-encoded proteins were removed from further analysis. The remaining nucleus-encoded proteins carry a phenylalanine within position 1–18 in 145 out of 187 cases. The 42 proteins lacking phenylalanine are predominantly low abundance proteins with a mean abundance of around 5 fmol on column, compared to 14 fmol on column average abundance calculated over all identified proteins. There are two ways to interpret these findings. First, it is possible that there are different import pathways that differ by their specificity. Since high abundance proteins are easier to detect, a potential detection bias for phenylalanine-containing transit peptides exists while low abundance proteins remained unidentified until recently (this study, Facchinelli et al., 2013). The second possibility is that the low abundance protein dataset is enriched for incorrect gene models, because more and better EST sequences are available for high abundance proteins.

### Analysis of non-F transit peptide containing proteins and examples for potential non-F precursors

To distinguish between these two possibilities, we analyzed the 42 proteins without phenylalanine at their N-terminus for additional evidence to support one or the other conclusion. The results of this search are summarized in Supplemental Table S3. In summary, we identified supporting EST sequences for 11 out of 42 non-F precursors. Of these, four ESTs supported the original gene model without F, while seven led to curation of the gene model. BLAST searches suggest a truncation for 15 out of the 31 remaining gene models.

In the set of proteins that do not carry F at their N-termini are three highly abundant opsins (Frassanito et al., 2010, 2013). Indirect immunofluorescence with antibodies against Cyanophopsin_1 in *C. paradoxa* cells showed labeling of the cyanelle envelope. Since knowledge about the targeting of proteins to the cyanelle envelope is missing, it may be hypothesized that phenylalanine is not required for the envelope-targeting of proteins. It is currently unknown whether a Toc75-related import channel is used for their translocation process. It has been speculated that the N-terminal phenylalanine attenuates the interaction with the cyanelle surface containing mono- and digalactosyldiacylglycerol, thus promoting contact with proteins present in the outer membrane such as a cyanelle Toc75 (Wunder et al., 2007). Therefore, it seems possible that at least some of the envelope-localized opsins are integrated into the membrane by a mechanism similar to the integration of AtOEP80, which contains the targeting information within its mature sequence (Inoue and Potter, 2004). The lack of a canonical transit peptide is supported by the identification of an unprocessed opsin with start position 2 in the TAILS experiment explaining the lack of F in the N-terminal region of these proteins (Supplemental Table 1).

Another non-F precursor is gene model 37109_cds:7008 that encodes for the 50S ribosomal protein L27. Many ESTs from this gene (e.g., EG947820.1) are shifted in their start 43 residues to the C-terminus compared to the annotated sequence. The EST-supported start MAHKKGSGSTKNGRDSN resembles the start of the mature part of the protein without any targeting signal. Interestingly, this N-terminal part of the sequence is 100% identical to the corresponding part of L27 of *C. merolae* where it appears to be encoded on the rhodoplast genome. Therefore, 50S ribosomal protein L27 might be imported into the cyanelle without any targeting signal. The only peptidoglycan-related protein found in this analysis is 54819_cds:11202 encoding UDP-N-acetylglucosamine-N-acetylmuramyl- (pentapeptide) pyrophosphoryl-undecaprenol N- acetylglucosamine transferase (murG), which is a stroma-localized peripheral inner envelope membrane protein. It appears to carry a targeting signal of about 80 residues without F when compared to bacterial proteins. A chaperonin GroEL homolog (37904_cds:7901), carries the aromatic residue tyrosine in the N-terminal region of the precursor (MEAAYVTSSP). When the GroEL precursor is compared to a homologous EST (EC658348.1) of the same species it appears that the latter carries a phenylalanine in the same position as the tyrosin (MDHAFVVP). It was shown that tyrosine can substitute for phenylalanine to some extent in *in vitro* import experiments (Steiner et al., 2005a). Thus, it is possible that F is functionally replaced by Y similar to the situation in diatoms where the aromatic amino acids phenylalanine, tryptophan, tyrosine allow efficient plastid protein import (Gruber et al., 2007).

The data presented here clearly support the prevalence of F in the N-terminal region of cyanelle transit peptides. The majority of the putative non-F precursors are represented by an incorrect gene model (Supplemental Table 3) and the abundant non-F cyanelle opsins do not carry a canonical transit peptide (see the TAILS data in Supplemental Table 1). Apart from the distinguishing N-terminal F, cyanelle transit peptides are similar in structure and amino acid composition to those of Arabidopsis, suggesting that basic principles guiding the protein translocation process into plastids were in place before the divergence of the three lineages after primary endosymbiosis.

### Conflict of interest statement

The authors declare that the research was conducted in the absence of any commercial or financial relationships that could be construed as a potential conflict of interest.
